# Extremely low frequency magnetic field distracts zebrafish from a visual cognitive task

**DOI:** 10.1038/s41598-025-90194-x

**Published:** 2025-03-12

**Authors:** Laura Ziegenbalg, Onur Güntürkün, Michael Winklhofer

**Affiliations:** 1 AG Sensory Biology of Animals, Institute for Biology and Environmental Sciences, Carl von Ossietzky Universität Oldenburg, 26129 Oldenburg, Germany; 2 Department of Biopsychology, Faculty of Psychology, Ruhr University Bochum, Universitätsstraße 150, 44780 Bochum, Germany; 3 Research Center Neurosensory Science, Carl von Ossietzky University of Oldenburg, 26111 Oldenburg, Germany

**Keywords:** Sensory pollution, Operant conditioning, Cross-modal interference, Magnetoreception, Wind farms, Sensory processing, Animal migration, Attention, Perception

## Abstract

Electromagnetic fields emitted from overhead power lines and subsea cables are widely regarded to be a disruptive factor for animals using the natural magnetic field as orientation cue for guiding their directed movements. However, it is not known if anthropogenic electromagnetic fields also have the potential to disturb animals attending to information from other sensory modalities. To find out, we trained adult zebrafish (*Danio rerio*) individually to perform avoidance behavior in response to a visual signal (green LED light spot), which in the exposure group was presented simultaneously with a sinusoidally changing magnetic field (0.3 Hz, group A: 0.015 mT, group B: 0.06 mT). Despite the salience of the visual signal, which was both sufficient and necessary to elicit conditioned avoidance responses, the 0.06 mT magnetic condition had a negative impact on learning performance and response behavior. This suggests that extremely low frequency technical magnetic fields of Earth strength amplitude can act as cross-modal distractor that diverts the attention of animals away from environmentally relevant cues based on nonmagnetic sensory modalities. Our research highlights the need to study the role of anthropogenic magnetic fields as sensory pollutant beyond the scope of magnetic orientation behavior.

**Supplementary Information:**

The online version contains supplementary material available at 10.1038/s41598-025-90194-x.

## Introduction

Many animals have been shown to possess a magnetic sense^1,2^, which enables them to use the Earth’s local magnetic field vector as a spatial orientation cue for navigation. In its basic form, this cue would indicate the local magnetic north direction, similar to a magnetic compass, while in a more advanced form it could serve as a guide to an animal to determine its position^3–10^. Regardless of the exact information that animals draw from the local magnetic field, it may no longer serve as a reliable spatial cue when warped or even masked by technical magnetic fields due to power lines and generators. This issue has become urgent with the increasing development of offshore wind energy and concomitant deployment of subsea cables for transmitting off-shore produced electrical energy to onshore grids^11,12^.

In order to investigate the potential effects of anthropogenic magnetic fields on marine animal species, numerous research studies have been conducted both in controlled laboratory environments^13–18^ as well as in the field^19–22^. Taken together, however, those studies yielded mixed effects. For example, Cresci and coworkers subjected fish larvae to a static magnetic field gradient ranging from 0.05 mT (ambient geomagnetic field) to 0.15 mT, as encountered near a high-voltage direct current (DC) subsea cable, and observed a reduced spontaneous swimming activity in haddock (*Melanogrammus aeglefinus*)^17^, but no effect on sand-eel (*Ammodytes marinus*)^18^. In a recent field study, smolts of Chinook salmon (*Oncorhynchus tshawytscha*) equipped with ultrasonic transmitters were tracked as they migrated across the San Francisco Bay toward the Pacific Ocean, but did not appear to be limited in their movements or migration success when crossing a DC carrying subsea power cable^21^. In an earlier biotelemetric study, six individuals of European eel (*Anguilla anguilla*, silver eel stage), ultrasonically tracked during their migration in the Baltic Sea, were found to significantly slow down (by ca. 25%) over a 130 kV alternating current (AC) power cable (140–300 Amps)^19^. Despite the ecologic value of these field studies, it was not possible to determine what environmental information the tracked animals actually used to guide their movements, and in particular, whether and where they may have relied on magnetic orientation/navigation.

A magnetically altered behavior in animals with magnetic sensory perception can also be the result of cross-modal interference, that is, when the process of recognizing, integrating, or memorizing cues in a non-magnetic sensory modality is interfered with by a sensory stream conveying unexpected magnetic field information. Cross-modal interference/distraction is a well-known phenomenon that also affects humans, for example when they read a text and simultaneously hear unexpected sounds or words that do not match the text (deviant distraction)^23,24^. Similarly, divided attention in a multitasking scenario, such as when we are on a phone call while driving, is likely to cause us to miss twice as many traffic signs as when we are not on the phone^25^. As a rule, cross-modal interference leads to poorer performance on the task in question or, in the case of dual-tasking, on one or both tasks^26,27^. More generally, a distractor can have effects at several cognitive levels, by.

i) Diverting limited attentional resources away from the learning task, thus lowering the learning rate;
ii) Increasing cognitive load, hence reducing the mental resources available for learning^28^, and.
iii) Interfering with the consolidation of information in memory, thereby diminishing the associative memory strength^29^.

A number of studies have observed cross-modal distraction of animals by anthropogenic acoustic noise, light pollution, or chemicals (see^30^ for review), but when it comes to anthropogenic electromagnetic fields (EMF), most work has focused on humans, but little on species in which a magnetic sense has been demonstrated. Not surprisingly, no consistent effects were found in studies that examined whether EMF exposure could affect cognitive and perceptual processing in human subjects^31,32^. In contrast, transient exposure to a 50 Hz EMF was found to cause a dose dependent reduction in olfactory learning of honeybees trained to associate an odorant with sucrose^33^. In that study the EMF was applied for 1 min after the end of each of five conditioning trials and thus within the assimilation phase of short-term memory in bees (< 60 s)^34^. The effects of EMFs on olfactory learning in honeybees can therefore be interpreted as distractive in the sense of ii) or iii), by providing task-irrelevant information that partially overrode the odorant/sucrose association just stored in the short-term memory.

**Fig. 1:**
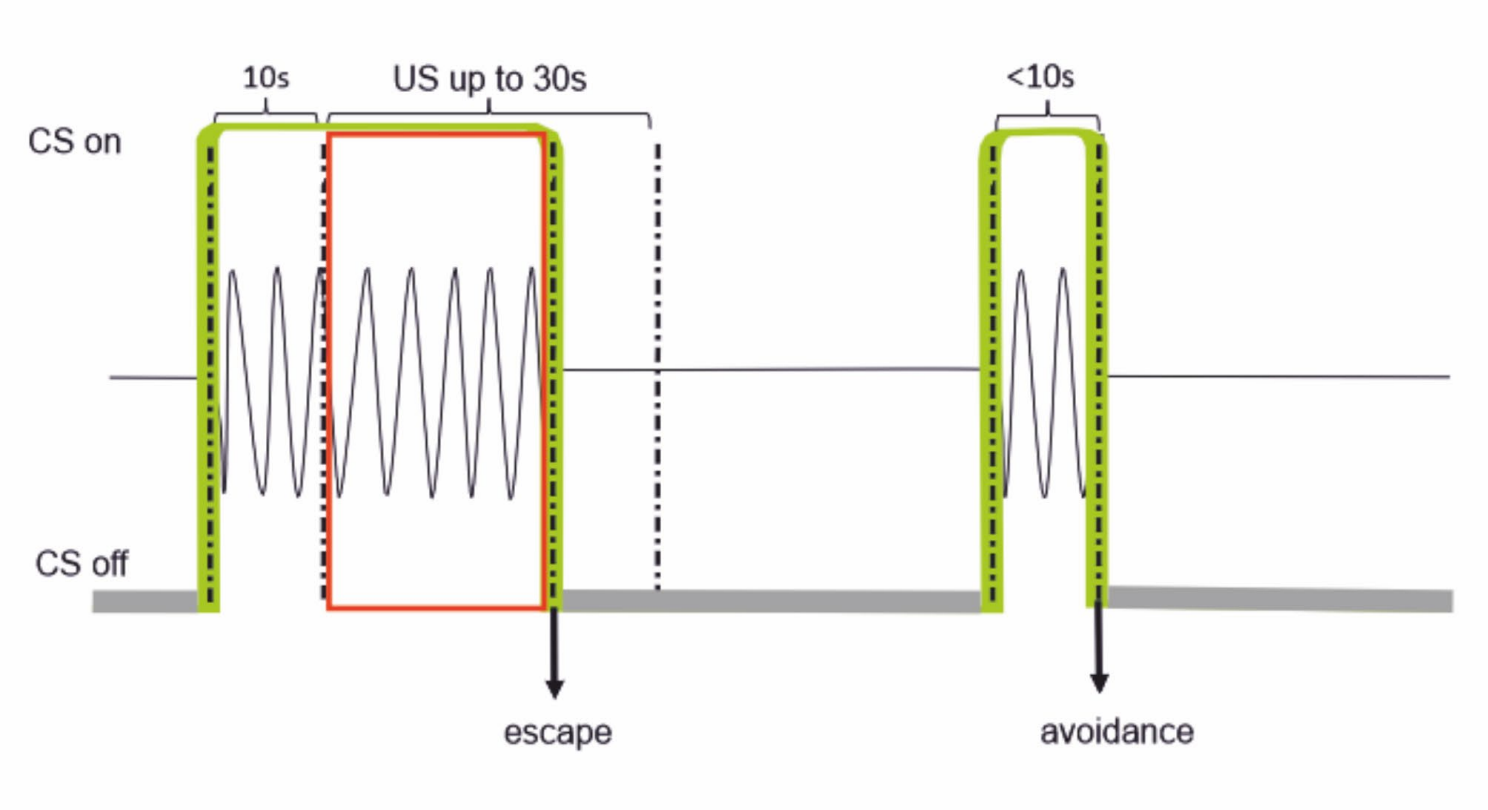
Conditioning scheme: A green LED light (L) and an oscillating magnetic field (M) are applied as compound CS at the onset of each trial. Left: If the fish stays in its compartment for more than 10 s, an aversive US (red) is applied, for a maximum duration of 30 s, in order to promote the desired response behavior, i.e., swimming into the other compartment. Both CS and US are terminated once the fish escapes or the 30 s are over. Right: No US is administered if the fish performs the desired response behavior within 10 s of CS presentation.

Here we studied if EMFs have the potential for cross-modal sensory distraction, specifically at the level of attention (i). To do so, we applied the EMF in the forward-conditioning process, simultaneously with a visual cue as conditioned stimulus (CS), using an avoidance learning paradigm with a mild electroshock as unconditioned stimulus (US) to be avoided by a timely conditioned response (CR) to the CS. We chose zebrafish (*Danio rerio*) as a magnetosensitive vertebrate model, one of the few species of fish in which magnetic orientation behavior (in the adult stage) has been demonstrated by at least three different research groups^35–38^. As a key vertebrate model for developmental biology, zebrafish were also used in studies of embryonic development under exposure to a 50 Hz electromagnetic fields (1 mT root-mean-square amplitude), reporting a delay in hatching when exposure had started just before hatching stage (48 h after fertilization), but not when it had started already in the 64-cell stage (2 h after fertilization)^39^. Long-term exposure to 1 mT@50 Hz was reported to have genotoxic or cytotoxic effects in larvae of other species of fish, such as rainbow trout (*Oncorhynchus mykiss*)^15^. The mechanisms underlying such effects are still elusive and may involve unspecific cellular processes that happen to be affected by a magnetic field, such as the cryptochrome-based circadian clock in fruit flies (*Drosophila melanogaster*), whose free-running rhythm period was found to become more variable by exposure to a 0.3 mT static magnetic field^40^. Likewise, electromagnetic field pulses delivered at a rate of 10 Hz were reported to modulate intracellular reactive oxygen species via a cryptochrome-dependent mechanism in both insect and mammalian cells^41^. Thus, to prevent such possible physiological side effects and to address primarily the magnetic sensory modality of zebrafish, we here used more gentle magnetic field exposure conditions, in the form of a slowly oscillating magnetic field (0.3 Hz oscillation frequency) and moderate oscillation amplitudes of 0.015 mT or 0.06 mT, superimposed on the static ambient magnetic field (ca. 0.05 mT). We deemed 0.3 Hz suitable as it is certainly slow enough to minimize magnetically induced electric artifacts. In contrast, had we used 50 Hz instead, then the induced electric field would not only be 50/0.3 = 167 times greater, but also could act as unwanted confounder by matching the 50 Hz carrier AC frequency of the electric field pulses we used as US. Moreover, we expected fish to be naïve for 0.3 Hz, which is distinctly different from any known electromagnetic frequencies (e.g., 50 Hz AC power grid frequency) which the fish in the rearing facility may have been pre-exposed to earlier without reinforcement, and which may have led to retarded response acquisition due to latent inhibition^42,43^. Specifically, we trained adult zebrafish to perform US avoidance in response to a compound CS ‘LM’ consisting of a visual signal (‘L’, green LED light spot, 570 nm wavelength) simultaneously presented with the oscillating magnetic field (‘M’, two exposure groups, A: 0.015 mT, B: 0.06 mT; control group 0 mT) during each learning trial (Fig. 1). In an earlier avoidance conditioning study on zebrafish with unisensory cues^44^, it was found that a visual cue was clearly more effective as a CS compared to a magnetic cue. Thus, we expected L to act as lead cue in the compound CS, engaging the fish in a visual task under simultaneous exposure to the anthropogenic magnetic noise. Using consistent criteria for learning and recall performance, we found fish to be cross-modally distracted by the 0.06 mT field, but not by the 0.015 mT field. All experiments were conducted in a Horner-type shuttlebox^45^ (SI Fig. 1a), placed in the center of a coil system (Si Fig. 1b), with fully automated stimulus application sequences and data acquisition procedures, including video and magnetic recordings as well as loggings of light-barrier activity (see Methods section for details).

## Results

### Control group (L+, no EMF exposure)

**Fig. 2:**
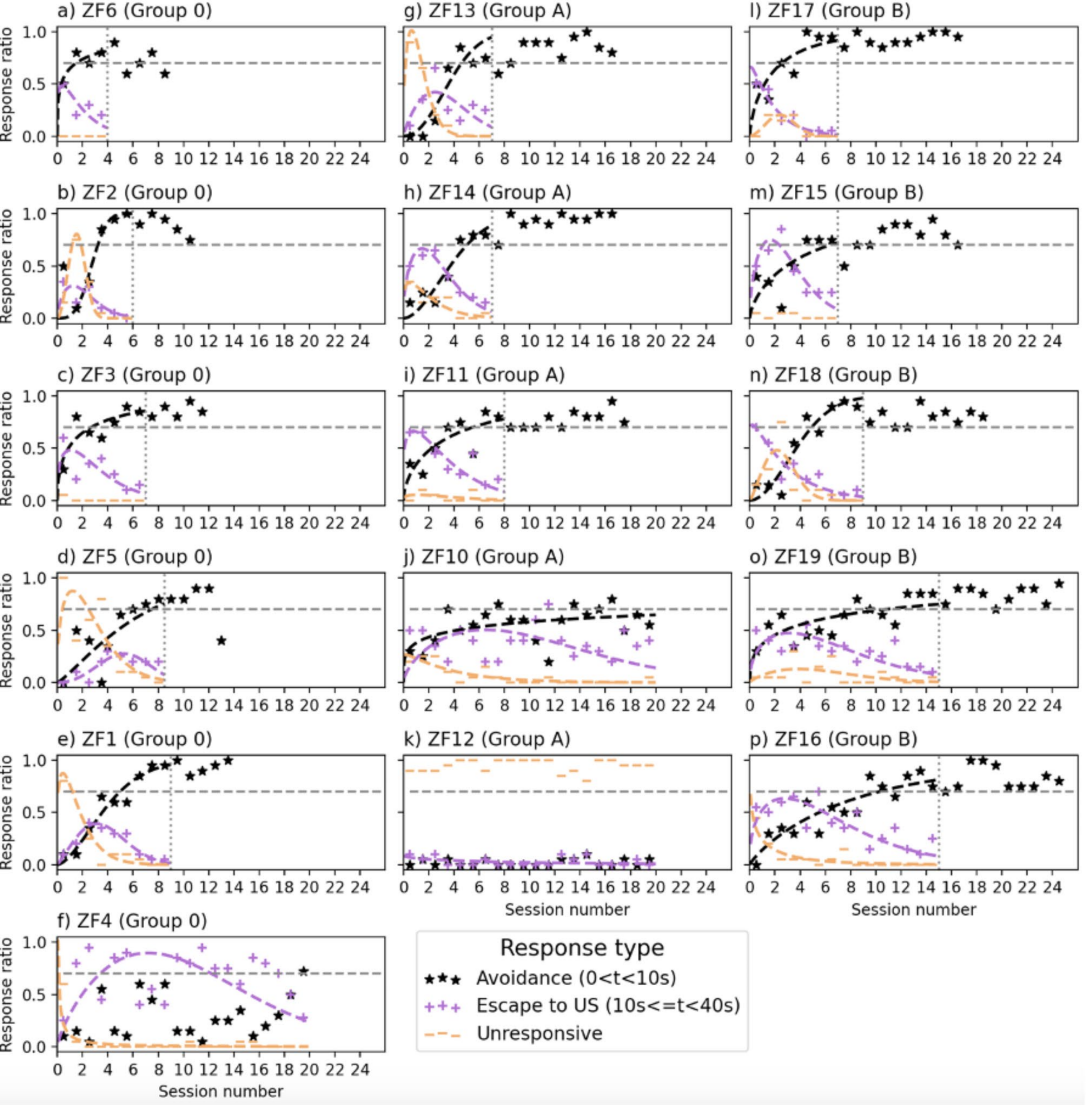
Learning and CS recall performance of individual zebrafish in avoidance learning paradigm with L as unisensory CS (**a**–**f**, left column) or LM as compound CS (**g**–**k**, middle column, group A: 0.015 mT;** l**–**p**, right column, group B: 0.06 mT). Each column is sorted according to individual learning rate (LC in Tables 1 and 2). The dotted horizontal line (grey) indicates the 70% threshold of correct avoidance responses, which had to be exceeded or at least maintained in three consecutive sessions (3 × 20 trials) to define successful learning. The vertical dotted line indicates the session number *LC* after which the learning criterion was reached and markers to the right of that line represent the ratio of correct avoidance responses in CS recall tests. The markers indicate the proportion (session mean) of responses of a given type (see legend). The black dashed line approximating the avoidance rate represents the learning curve, the other two lines approximate the rates of non-responses (orange) and US escape responses (magenta).

In the initial reinforced learning trials, fish showed neither freezing nor escape behavior upon presentation of the LED light spot (L), which suggests that adult zebrafish fish did not have a prior negative association with it. Thus, L could act as a neutral stimulus that could turn into a conditioned stimulus (CS) in the course of the avoidance learning paradigm. This contrasts with the innate negative phototaxis observed in zebrafish larvae^46^, which would have led to escape behavior even without negative reinforcement through the aversive unconditioned stimulus (US). Overall, five out of six naïve individuals of the control group learned to avoid the US by performing the conditioned response (CR) within 10 s after presentation of L. They required between five and ten L + training sessions to reach the predefined learning criterion (see Table 1 for summary). As can be seen in Fig. 2a–f; Table 1, some fish had hyperbolic learning curves, with highest learning rate in the beginning ($\:0<{S}_{\text{av}}<1)$, whilst others had sigmoidal learning curves ($\:{S}_{\text{av}}>1),$ with a steep increase after several sessions of latency in which these fish failed to perform the escape response to the US (see orange traces labelled “unresponsive behavior” in Fig. 2). This suggests that fish with sigmoidal learning curves perceived the US as less aversive compared to fish with hyperbolic learning curves.

**Table 1:** Unisensory learning and CS recall performance of all individual fish from group 0 (LED signal) that reached the predefined learning criterion within 20 sessions.

Group	Fish ID	S_av_	L_av_ (sess.)	LC (sess.)	CS recall rate (100 L: L trials)	FPR (10 null trials)
0	a) ZF6	0.4	0.9	4	0.72	0
0	b) ZF2	3.3	3.0	6	0.89	0
0	c) ZF3	0.5	1.8	7	0.86	0.2
0	d) ZF5	1.2	6.4	8.5	0.76	0.1
0	e) ZF1	1.6	4.5	9	0.94	0.2
Mean (*n* = 5) :				*6.9*	*0.83*	*0.1*

*S*_av_, *L*_av_: shape and scale parameter of Weibull curve (see Eq. 2) fitted to learning curves of avoidance behavior, shown in Fig. 2 (a–e, black curves); *LC*: session number at which pre-defined learning criterion was reached, with each session comprising 20 trials (except for session 5 of ZF5, which had only 10 trials); CS recall rate: proportion of correct responses in trials with CS presented; *FPR*: ratio of false positive responses in sham trials.

Mean values in italic.

**Table 2:** Learning performance of individual fish and response performance in multisensory recall tests with compound CS (LM: LM) and in unisensory split-cue discrimination tests (LM: L, LM: M).

Group	FishID	S_av_	L_av_ (sess.)	LC (sess.)	CPR (LM: LM)	FPR	CPR (LM: L)	CPR (LM: M)	*p*-value CPR(LM: M) vs. FPR
A	g) ZF13	2.2	4.2	7	0.84	0.27	19/20	4/13	0.48
A	h) ZF14	1.8	4.5	7	0.94	0.19	20/22	2/12	0.70
A	i) ZF11	0.6	4.1	8	0.78	0.33	11/16	3/12	0.81
A	j) ZF10	0.25	17.0	(> 20)					
Mean (*n* = 3):				*7.33*	*0.85*	*0.26*	*0.86*	*0.24*	
B	l) ZF17	0.7	2.0	7	0.91	0.35	28/28	6/15	0.44
B	m) ZF15	0.7	4.9	7	0.79	0.14	14/18	0/17	1
B	n) ZF18	1.9	4.5	9	0.79	0.36	21/23	5/14	0.61
B	p) ZF19	0.4	6.8	15	0.80	0.20	24/26	4/15	0.35
B	q) ZF16	0.9	8.4	15	0.82	0.06	20/20	4/16	0.013
Mean (*n* = 5):				*10.6*	*0.82*	*0.22*	*0.91*	*0.25*	

Magnetic field oscillation amplitude was 0.015 mT (group A) and 0.06 mT (group B). *S*_av_, *L*_av_ shape and scale parameter of Weibull function (Eq. 2) fitted to avoidance responses in learning sessions, shown in Fig. 2 (g–i and l–p, black lines); *LC*: session number at which learning criterion was reached, with each session comprising 20 trials; *CPR* (LM: LM): proportion of correct response in tests with compound CS presented (CS recall rate); *FPR*: false positive rate (spontaneous shuttling rate); *CPR*(LM: L) and *CPR*(LM: M): proportion of correct positive responses in unisensory tests with either L or M presented, respectively. p-value: binomial test with the one-sided alternative hypothesis *CPR(LM: M)* > *FPR*.

Mean values in italic.

After reaching the learning criterion, the fish of the control group kept their CR in CS presentations without reinforcement and had L: L recall rates between 72% and 94% (see Table 1). In sham trials, the proportion of false positive responses amounted to between 0% and maximally 20% with an average of 10% over five individuals. This is much lower than the proportion of correct positives in CS recall trials (*p* < 10^− 4^, proportion test). Thus, it is likely that the animals’ response behavior was not due to random shuttling by an overall increase of arousal-based swimming activity while trying to avoid the US. To corroborate the statistical inference, we inspected video recordings which revealed a clearly discernible shuttling response to the CS which qualitatively differed from the relaxed swimming in intertrial intervals or sham trials. We found the recall performance to sustain over all five test sessions conducted (i.e. over five consecutive days), which all together confirms the robustness of the CR.

### Exposure groups (LM+)

Eight out of ten naïve fish of the magnetic field exposure groups (LM+, group A: 0.015 mT, group B: 0.060 mT) acquired a CR to the compound CS ‘LM’ (3/5 in group A, 5/5 in group B, no significant difference between groups, proportion test), but again differed in their individual learning performance and learning-curve characteristics (see Table 2; Fig. 2g–p). Significantly, fish of group B on average took 10.6 sessions to reach the pre-defined learning criterion, which is ca. 50% longer than those of the control group 0 (6.9 sessions) (P value = 0.05, see Table 3 for detailed report of glm). One individual of group A (ZF10) was on a learning trajectory that we extrapolated to pass the criterion not before session 38. Another individual of group A (ZF12) remained persistently unresponsive to US exposure, suggesting that the electric stimulation was not perceived as aversive at all and thus ineffective for negative reinforcement.

**Table 3:** Summary of general linear model (GLM) testing learning performance across the three groups in terms of sessions required to reach learning criterion (see Tables 1 and 2 for individual values).

Group	Estimate	Std. error	z value	Pr(>|z|)
0	1.9315	0.170	11.35	
A	0.0609	0.273	0.223	0.82
B	0.4293	0.219	1.963	0.0497 (*)

Note that GLM for poisson error model reports log transformed estimates. The arithmetic mean of group 0 (defining the intercept) is exp(1.9315) = 6.9. The estimate of the learning rate for group B is exp(0.4293) = 1.536 times the value for the control group, i.e., ca. 54% larger ($\:\text{p}\approx\:0.05$), and that of group A is exp(0.0609)=1.06 times that of the control group (ca. 6% larger, not significant, $\:p\approx\:0.8$). Residual deviance was 8.85 on 10 degrees of freedom.

*significant (*p* < 5% significance).

**Fig. 3:**
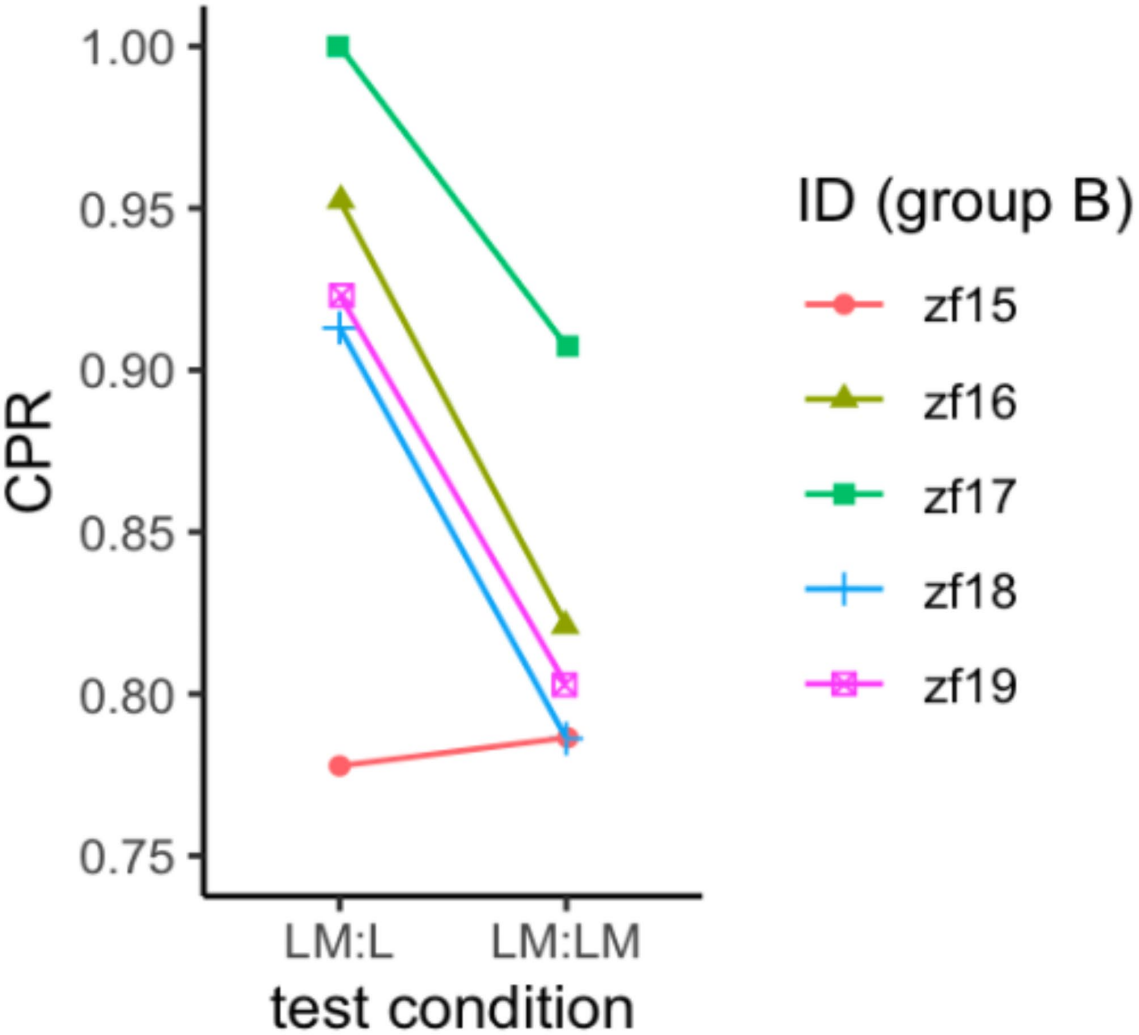
Distractive effect of 0.06 mT magnetic field stimulus M in test sessions of group B fish trained to the compound cue LM. Correct response rate (CPR) to light stimulus only (LM: L) compared to light stimulus with magnetic stimulus M superimposed (LM: LM). See Table 4 for statistical analysis.

Once they had reached the learning criterion, Group A/B fish kept up their CR to the compound CS over the ten LM: LM test sessions conducted without further reinforcement. Individual CR recall rates in LM: LM trials ranged between 71% and 94% (see Table 2). Given the multimodal nature of the compound CS, we wanted to know if unimodal presentation of either the visual or magnetic signal is already sufficient to elicit the conditioned response and therefore placed a few split-cue trials (LM: L and LM: M) at random positions in each CS recall-test session. All eight individuals consistently responded to L, at a much higher rate (*CPR* (LM: L) between 80% and 100%, see Table 2) compared to the false positive rate (FPR between 3% and 35%, see Table 2), which was determined by counting spontaneous shuttling in intertrial intervals. Seven of the eight fish did not respond significantly more often to the magnetic signal alone (LM: M) compared to FPR and even the one fish (ZF 16) that stands out in Table 2 with a p-value less than 0.05 only responded in 4 out of 16 LM: M trials, but without showing a distinct response behavior. Most strikingly, response rates in group B were significantly higher in LM: L than in LM: LM trials (Fig. 3, odds ratio = 2.5, P value < 0.01, see Table 4 for detailed report).

**Table 4:** Summary of mixed-effects model testing response rates of group B fish trained in compound CS and tested in two conditions (LM: LM vs. LM: L trials), using a binominal error model, with condition as fixed effect and zebrafish id as random effect.

Condition	Estimate	Std. error	z value	Pr(>|z|)
LM: L	2.487	0.374	6.66	
LM: LM	-0.934	0.359	-2.60	0.009 (**)

Estimates are reported in terms of log-odds, i.e., log(rate/(1-rate)). The estimate of the response rate is 1/(1 + exp(-2.487)) = 92.3% for condition LM: L and 1/(1 + exp(- (2.487–0.934))) = 82.5% for condition LM: L.

**very significant (*p* < 1% significance).

To summarize our key results, in both magnetic noise exposure groups, CRs were elicited in LM: LM and LM: L trials, but not in LM: M trials. Thus, L in fish conditioned to LM is both sufficient and necessary for evoking a CR. Most importantly, the fish in group B (0.06 mT) were distracted by M, as evidenced by significantly lower response rates in CS recall tests (LM: LM) compared to split-cue (LM: L) trials, as well as by slower learning progress compared to fish of group 0 and A.

## Discussion

So far, extremely low-frequency electromagnetic fields (EMFs) have been regarded as a disruptive factor for animals performing magnetic orientation behavior. In contrast, little consideration has been given to the possibility of cross-modal interference by EMFs with more common behaviors such as prey detection, predator recognition, or social interactions, all of which involve the processing of multisensory streams from non-magnetic stimulus modalities. Our results clearly show that short-term magnetic field oscillations of 0.06 mT (but not 0.015 mT) have a negative impact on learning performance and response rates in a visual task. This tallies with a Pavlovian conditioning study on honeybees, whose olfactory learning performance was found to be impaired by transient 50 Hz EMF exposure of 0.1 mT and higher, but not 0.02 mT^33^. In terms of conditioning procedure, the key difference between our study and that of Shepherd et al.^33^ was the timing of the transient EMF exposure in relation to the CS-US pairings (simultaneous vs. delay) and thus the cognitive process affected by the EMF distractor (attention vs. short-term memory consolidation). Given the ease with which the proboscis extension reflex can be trained in bees, it should be straightforward to also test for distraction at the level of attention, by applying the EMF in the forward conditioning, simultaneously with the CS. Conversely, the operant conditioning paradigm developed here for zebrafish can be modified easily to study effects of delayed EMF exposure on short-term memory consolidation.

### Salience of visual signal

The visual signal L in the compound CS was designed as lead cue to engage the fish in a visual learning task. L turned out to be so salient that it completely overshadowed the magnetic signal M, which in turn never became a CS but acted as distractor instead. Overshadowing has long been known from classical conditioning experiments (p. 141–143 of ref^47^), particularly from illness-induced aversions to drinking flavored dyed water, where the visual cue (dye) in the combined CS was so salient for quails that they did not respond to the redundant chemical cue (flavor), and vice versa for rats^48^. In pigeons trained to an auditory plus visual compound CS, the relative salience of cues depended on the nature of the task, with the visual cue dominating over the auditory cue in an appetitive task, and vice versa for an avoidance task^49^. In most overshadowing experiments, the effect of the salient component tested singly seemed to be equal to that of compound and this was indeed the case for the effectively neutral 0.015 mT condition (group A fish). In contrast, for the distractive 0.06 mT exposure condition (group B fish, Fig. 3), we observed a consistently higher response rate to the salient cue (LM: L) compared to the compound (LM: LM). We note that the influential Rescorla and Wagner model^50^ of classic (Pavlovian) conditioning would predict the opposite, i.e., that the response rate for LM: LM should be at least as high as for LM: L (see Appendix). However, the Rescorla and Wagner model is known to fail in learning contexts where one stimulus clearly dominates over the other^51^, as in the illness-induced drinking aversions. Also, the model does not include attention as an explicit variable, while we suggest that the two stimuli primarily competed for attention. That is, fish focused their attention primarily on the visual cue as a salient predictor for the US during learning. Since attention defines which neural systems gain activity strength^52,53^, this system is also concomitantly able to alter its associative strength with the CR^54,55^. As a consequence, the magnetic sensory modality gained less memory strength or was possibly even filtered out to prevent distraction from the visual cue. But once our fish started to learn the association between L and US, they did get distracted by the M cue every so often, thereby missing the L cue, which reduces the effective number of L learning trials per session and thus increases the total amount of compound trials required to reach the learning criterion. The distractive effect of M must have persisted in LM: LM tests, as evidenced by the lower response rates compared to LM: L tests (Fig. 3).

### Preparedness

The dominance of the visual cue over magnetic cues in our avoidance task can be explained in terms of predisposition or “preparedness”^56^ for certain cue-to-consequence dyads. Fish – and in particular small prey fish such as zebrafish – are evolutionarily well prepared to associate an abrupt change in the visual modality with an adverse event (e.g., a looming predator) and to integrate it in the neural process of making the decision to escape. In their natural environment, i.e., in silt-bottomed, well-vegetated pools and rice paddies in India^57^, zebrafish may use the magnetic field for directed swimming while escaping from predators^35^. However, the very decision to escape is triggered by salient cues detected in visual or mechanical sensory modalities. In general, sudden changes in the visual, mechanical, chemical, or thermal sensory modalities are often indicators of new environmental situations that can elicit fear and necessitate adequate response behaviors. In contrast, the Earth’s magnetic field does not undergo rapid changes (even in a strong geomagnetic storm, the field intensity fluctuates by not more than ca. 1%), which is why it can serve as temporally stable cue for spatial orientation. From this point of view, there is no obvious reason why animals would have evolved a neural circuitry that would make them prepared to perform – in response to a changing magnetic field – a distinct behavior other than adjusting their movement direction. Such a biological constraint of learning could explain the many unsuccessful attempts at conditioning birds to magnetic field changes^58^, even in species, such as European robins^59^, that are known to use the Earth’s magnetic field for orientation in absence of spatial cues on other sensory modalities. On the other hand, several species of fish have been shown to be amenable to unimodal magnetic conditioning, irrespective of the paradigm, be it Pavlovian fear conditioning (Japanese eel^60^, rainbow trout^61,62^), be it operant conditioning with positive reinforcement (Yellowfin tuna^63^ and rainbow trout^44,64^) or negative reinforcement (Mozambique tilapia^44^), which altogether demonstrates that fish can integrate magnetic percepts in reward and fear circuits. This suggests that when it comes to learned responses to magnetic fields, fish are apparently not subject to the same biological constraints as birds are. However, given the significant fraction of the teleost fish brain that is involved in processing visual information^65,66^ and given that predatory fish like tuna, trout, and tilapia rely largely on vision (as indicated by e.g. the observation that foraging efficiency is reduced in turbid waters^67^), it is reasonable to assume that they are better predisposed for integrating visual rather than magnetic percepts into the process of making a decision to move. We therefore predict for these predatory fish as well that visual cues will overshadow magnetic cues when offered simultaneously as predictors for an US.

Our observation that the magnetic component of the multimodal CS did not become a CS on its own does not contradict the body of successful *unimodal* magnetic conditioning attempts on fish mentioned above and, in particular, does not imply that zebrafish could not be trained to respond to it in a *unimodal* magnetic conditioning attempt, which we did not embark on here. On the contrary, our results are consistent with an earlier unimodal avoidance conditioning study on zebrafish^44^, where a visual signal was clearly more effective as a CS than was a magnetic one, which makes the visual cue the easier cue to learn about in the multimodal compound CS.

### Individual differences in learning performance

The fish in our paradigm showed considerably interindividual variability in learning rates and in tolerance to the US, which could be overcome in future studies with a higher US strength, e.g., 0.73 V/cm^68^, or even 1.5 V/cm^69^, compared to 0.2 V/cm administered here. Some individuals (ZF10 in group A, ZF16 and ZF19 in group B) apparently had more difficulties than others in filtering out the noise on the magnetic sensory modality when attempting to attend to the visual cue. This may reflect variability at the sensory and/or cognitive level, and in this respect it is interesting to note that zebrafish personality was suggested to influence responses to or weighing of magnetic sensory inputs, based on the experimental observation that “reactive” (i.e., shy) personalities had delayed rheotaxis responses curves when the static magnetic field pointed upstream compared to downstream, while “proactive” (i.e., exploratory) personalities had undelayed rheotaxis responses curves regardless of magnetic field polarity^70,71^. For further studies into magnetic behavior of zebrafish, it may therefore be useful to adopt the practice^71^ of categorizing individuals in terms of personality ahead of the experiment. In any case, in conditioning studies focused on sensory feats, it is not ideal to train a shoal of fish, which may comprise influencers and followers or related personality dichotomies, e.g. proactive and reactive personalities^71,72^, key fish and non-key fish^73^, as well as low or high performers^74,75^. An obvious problem with shoal conditioning is that the US is terminated or avoided for the whole group as soon as the first individual of the shoal performs the desired behavior (escape or avoidance), thereby hindering the others from learning the desired conditioned response by the outcome of their own actions. Also, when it comes to the statistical analysis of shoal behavior, the appropriate choice of sampling unit for replication is not clear (i.e., whole shoal versus individual animals)^76^. This was shown^77,78^ to be a particularly problematic issue in magnetic association experiments with fruitflies^79^, which had been tested in groups of ca. 100 individuals each and then were analyzed as if each individual in a group had acted as an independent biological replicate. This high level of pseudoreplication led to inflated statistical significance of a magnetic preference behavior, which in a replication attempt turned out to be irreproducible^77^.

### Magnetic sensory mechanism

The distractive effect of the magnetic field exposure we observed in our individually conditioned zebrafish study strongly suggests that they are capable of magnetic sensory perception, in line with previous magnetic orientation studies on zebrafish^35–38^. This raises the question about the type of magnetic field detection mechanism involved. Based on their different compass orientation behavior under darkness vs. white light, zebrafish were suggested to have two magnetic sensory pathways^38^, one based on magnetic particles sensitive to the magnetic field direction and responsible for polar compass responses in darkness^38^, the other on a radical-pair mechanism sensitive to the orientation of the magnetic field axis and responsible for axial compass responses under white light^35,37,38^. A hot candidate sensory protein for the radical-pair mechanism is the flavoprotein cryptochrome 4 (CRY4), which under illumination with shortwave light (UV to blue) can form a magnetically sensitive radical pair, as experimentally demonstrated on erCRY4 (European robin) in vitro^38^. Zebrafish (zf) CRY4 and erCRY4 share a high degree of molecular similarity^81^ and have similar UV-VIS absorption spectra^82^, with zfCRY4 being the first vertebrate cryptochrome found to have a stably bound flavin in a semiquinone (radical) state after illumination with short-wavelength light^82^. Moreover, zf*CRY4* mRNA was reported to be highly expressed in the zebrafish retina^83,84^. However, the green LED light used in our multisensory stimulus (against near-infrared background illumination) is very unlikely to produce radical pairs in CRY4 as the LED wavelength of 570 nm is outside the in vitro absorption spectrum of CRY4, falling off strongly at about 500 nm^82,85,86^. We therefore suggest that the conditions in our LM + training trails stimulated the magnetite-based pathway, probably in the trigeminal system^61,87^, in association with which magnetic particles have been reported in other species of fish^88–92^ (but see^93,94^). A third yet unlikely possibility for a magnetoreception pathway in zebrafish would be through electromagnetic induction with inner-ear semicircular canals as accessory organ ^95^, which has long been considered impossible in animals that are not equipped with highly sensitive electroreceptors^96^, but has recently been suggested as a feasible mechanism for homing pigeons^97^. The sequence data of the voltage-sensitive calcium channel Ca_V_1.3^97,98^ suggest that zebrafish, as opposed to pigeons and skates, do not have the crucial amino-acid motif that in^98^ was shown to confer the low voltage threshold to skate Ca_V_1.3. Unless zebrafish have a different voltage-gated ion channel of similar sensitivity, they are highly unlikely to detect the magnetic signal by electromagnetic induction. After all, the induced electric field along a semicircular canal (7 mm diameter) due to the sinusoidally oscillating magnetic signal (0.06 mT amplitude at 0.3 Hz) is only ca. 2 nV/cm, representing an induced voltage of 4.5 nV. For comparison, the detection threshold reported for fish equipped with highly sensitive ampullae of Lorenzini is 5 nV/cm ^98^. Thus, we consider it highly unlikely that the oscillatory magnetic field used here presented an adequate stimulus for an electromagnetic induction mechanism. One may object that the fish could make body turns to actively use the induction mechanism, in which case however it would only be sensitive to the horizontal component of the field, which stayed constant in all experiments and therefore did not act as a stimulus.

### Conclusions

We have shown that anthropogenic magnetic noise with 0.06 mT amplitude can act as a cross-modal distractor that diverts the attention of magnetically sensitive animals from perceiving environmentally relevant cues in nonmagnetic sensory modalities. Anthropogenic magnetic noise may therefore impact on animals capable of magnetoreception in more ways and modality dimensions than has been recognized thus far. It should be regarded as a *bona fide* sensory pollutant, similar to anthropogenic chemicals, light pollution, and acoustic noise^30^. Critically, 0.06 mT is 1000 times lower than the safety limit for public exposure, which is solely based on electromagnetic induction effects (e.g. 57 mT amplitude or 40 mT RMS at 1 Hz, see^99^ ). To put this in perspective, 0.06 mT, or 47.75 A/m, corresponds to the magnetic field generated by a single-phase current of 300 Amp at a distance of 1 m from the power line (Biot-Savart law). On the positive side, we found no measurable effect on learning and performance at 0.015 mT, which suggests that fish can effectively filter out moderate magnetic noise levels when engaging in a task directed toward a salient cue. We thus consider 0.015 mT a relatively safe level for occasional exposure of magnetosensitive animals outside the scope of magnetic orientation behavior, pending further studies on different species, particularly on long-distance migratory fish such as salmon and eel, whose magnetic sense is deemed more sensitive and thus likely to be more susceptible to EMF interference than that of zebrafish.

## Materials and methods

### Animals

Adult zebrafish (wildtype) were obtained from a commercial breeding facility in Germany and kept separately in a 3-liter plastic aquarium each, at 26 °C under a 14 h:10 h light: dark cycle during the whole course of the study. Fish were fed twice daily, alternating between commercial fish food (Züchterflocken mit Spirulina-Algen, FiMö Aquaristik GmbH, Bünde, Germany) and 2 day old *Artemia salina* (Artemix, HOBBY, Dohse Aquaristik, Gelsdorf, Germany).

All animal procedures were approved by the Animal Care and Use Committees of the Lower Saxonian State Office for Consumer Protection and Food Safety (LAVES, Oldenburg, Germany, Az: 3319-02502-04-17 2721). All experiments were carried out in accordance with the approved guidelines. This study conforms with the guidelines recommended on https://arriveguidelines.org.

### Setup

We designed a fully-automated Horner-type shuttlebox (Fig. S1), similar to what has been commonly used in aversive conditioning of fishes^45,100,101^. The shuttlebox (12 L; 30 cm length, 20 × 20 cm height) was built from nonmagnetic materials (acrylic glass and PVC) and consisted of two compartments (13 cm × 12 cm × 20 cm height), separated by a hurdle 5 cm wide, 3 cm long, and 11 cm high, with its upper surface 1 cm beneath the water level (see Fig. S1 for illustration). To automatically detect shuttling of a fish between the two compartments, the shuttlebox was equipped with a custom-built infrared light barrier reaching from 0 cm to 2 cm above the hurdle.

Each compartment was equipped with an electrically independent pair of stainless-steel electrode plates (13 cm long, 20 cm high) fitted to each side in parallel orientation with a plate-to-plate distance of 12 cm. The plates were configured to produce an electric field of ca. 0.2 V/cm (2.5 V across 12 cm) as US. The US consisted of a 10 msec long sequence of 5 electric field pulses (50 Hz carrier frequency) separated by 10 msec interpulse interval (50% duty cycle). To administer light (e.g., as conditioned stimulus), a small green LED (570–575 nm wavelength) was placed at the far end of each compartment to illuminate one or the other compartment selectively.

The shuttlebox was placed in the center of a two-axis square Helmholtz-coil system, consisting of copper wire (1 mm diameter) wrapped in 19 turns on a 90-cm edge length aluminum frame in a double-wound configuration^102^ allowing for sham magnetic field control conditions where current flows in both windings but in opposite sense, thereby producing no net coil field. To deliver an oscillatory magnetic field signal, we fed the vertical coil axis with a sine-wave current ($\:f=0.3$ Hz oscillation frequency) produced with an analog signal generator, resulting in a periodic modulation of the inclination angle and total intensity of the background magnetic field ($\:{B}_{z,0}=0.0387$ mT total field intensity, 66 degrees inclination), without affecting the declination angle. The total signal thus is given by

1 $$\:{B}_{z}\left(t\right)={B}_{z,0}+{B}_{f}\text{sin}\left(2\:\pi\:\:f\:t+{\varphi\:}_{0}\right),\:$$

where $\:t$ is the time since start of a session, $\:{B}_{f}$ is the signal amplitude (0.015 mT or 0.06 mT in trials of group A or B, resp., and 0 mT in intertrials), and $\:{\varphi\:}_{0}$ is the initial phase of the oscillation. As trial onsets were picked by a random generator and thus not phase controlled, the effective phase present at the start of any given trial was random too, i.e., the field started anywhere on the ascending or descending slope of the sine curve. Yet, there were always three full oscillation periods in the interstimulus time of 10 s between CS and US.

The effective total field intensity $\:\left|B\left(t\right)\right|$ oscillated between 0.025 mT and 0.053 mT for 0.015 mT signal amplitude and between 0.015 mT and 0.097 mT for 0.06 mT signal amplitude. The inclination oscillated between 52 deg and 72 deg for 0.015 mT signal amplitude and between − 57 deg and 80 deg for 0.06 mT signal amplitude. In the latter condition, the inclination is negative for 1 s during each 3.33 long signal period. For calibration of the magnetic field stimulus, we used a three-axis fluxgate magnetometer (Institut Dr. Foerster GmbH, Reutlingen, Germany) positioned in the center of the setup. For monitoring purposes during experimental sessions, we placed the magnetometer beneath the setup.

The coil system containing the experimental setup was covered with black cloth to prevent animals from perceiving outside cues. Since zebrafish show no responses in behavioral assays using 950 nm IR-light^103,104^, the shuttlebox was illuminated from below with a custom-built infrared (IR) light Tables (940–950 nm) to enable observation of the fish from above with an IR-sensitive camera (Raspberry Pi NoIR Camera V2 connected to Raspberry Pi 3, Model b, RS Components Ltd and Allied Electronics, United Kingdom).

The shuttlebox was controlled by a microcomputer, which delivered the conditioned stimulus (CS) as well as the unconditioned stimulus (US) in case no crossing was detected within 10 s after the presentation of the CS. Furthermore, the registration of light barrier detection events was synchronized with the stimulus administration protocol to ensure correct timings.

### Experimental procedure

To minimize residual levels of odors or stress hormones in the experimental setup, the water was replaced with fresh water from the source that supplied the zebrafish housing tanks. The water level above the hurdle was 9 +/- 1 mm, i.e., high enough to permit the animal to cross easily from one compartment to the other but low enough to discourage randomly crossing over the hurdle. The fish was allowed to adjust to the shuttle box for fifteen minutes before the session started.

#### Training

Each naïve individual was trained in two sessions per day, with a resting interval of 5 h between the sessions and an acclimatization phase of 15 min in the shuttle box before each session. A training session comprised 20 trials. Each training trial (see Fig. 1 for conditioning paradigm) starts with a continuous presentation of an initially neutral signal that is to become the CS. The visual signal was green LED light (L) of constant intensity, always delivered to the compartment where the fish was in that moment. The compound signal was a sinusoidally oscillating magnetic field (MF) applied simultaneously with the green LED light. At the very moment the zebrafish crossed the barrier, the signal was shut off, and the trial ended. If crossing occurred within 10 s after the onset of the signal, the trial counted as successful avoidance response and no negative reinforcement ensued. In contrast, no crossing within 10 s entailed a negative reinforcement in the form of weak electric shocks of 0.2 V/cm amplitude (US). Crossing during presentation of the US counted as escape response. If still no crossing occurred during 30 s long presentation of the US, the trial was discontinued and counted as non-response. Each trial was followed by a neutral inter-trial interval whose length was chosen randomly between 60 s and 180 s to ensure an aperiodic pattern in the sequence of trials. The animals were trained until they reached the learning criterion, defined as three consecutive sessions, each with at least 14 correct avoidance responses out of 20 trials, as in refs^101,105^. If an animal had not reached the criterion in up to 20 sessions, it did not advance to the recall performance tests.

#### Compound CS recall tests and unisensory split-cue tests

Once an animal had reached the learning criterion, its ability to recall the learned behavior without negative reinforcement was tested in *n* follow-up sessions (*n* = 5 for group 0, *n* = 10 for group A and B). Each test session comprised 20 trials with the CS presented, i.e., the L signal for group 0 (L: L trials) and the compound (LM) signal for group A and B (LM: LM trials). For group 0, we additionally placed two sham trials in each test session at random positions during which no CS was presented and that we used to count false positive crossings, to be compared with correct positive responses upon CS presentation. For group A and B, a few unisensory split-cue tests (LM: L and LM: M) were placed randomly in between the multisensory CS sessions, to determine the relative salience of the two cues and to find out if response rates and times differ between multisensory and unisensory tests. As with training, crossing within 10 s after the onset of a signal (LM, L, or M) counted as correct response and any two test trials were separated by an inter-trial interval of random length (between 60 s and 180 s), which served us to determine the number of spontaneous crossings (false positives) when no signal was given.

### Statistical analysis

#### Modelling of individual learning curves

As demonstrated earlier^106^, the rate of learning should be determined for each individual separately. As a parametric learning curve, we use the Weibull distribution,

2 $$\:F\left(t;L,S\right)=1-\text{exp}\left(-{\left(\frac{t}{\:L\:}\right)}^{S}\right),\:$$

where the variable $\:t$ is the cumulative trial number, $\:S$ is the shape parameter, and $\:L$ is the scale parameter so that at $\:t=L$, $\:F$ amounts to $\:1-\frac{1}{e}=0.63\:$(63% correct response rate) and has a slope (learning rate) of $\:\frac{S}{eL}$. Note that $\:F\left(t;L,S=1\right)$ is simply the exponential distribution function, characterized by a constant learning rate parameter $\:\beta\:=\frac{1}{L}$.

For each individual fish, the parameters $\:{L}_{\text{av}}$ and $\:{S}_{\text{av}}$ are determined by fitting $\:F\left(t;L,S\right)$ to the binary sequence of avoidance responses during the learning sessions. To model transient behavior during learning (i.e., escape responses and non-responsive behavior), we fitted a Weibull distribution to the respective cumulative distribution of responses, taking advantage of the observation that these cumulative response curves saturate when approaching the learning criterion, which allows for precise fitting. We then use the derivative of the fitted curve, $\:\frac{dF}{dt}\left(t;L,S\right),$ (which is the probability density function of the Weibull distribution) to plot transient response behavior in Fig. 2. All curve fitting was performed with the function curve_fit from the python module scipy.optimize.

#### Statistical testing of learning performance at group level

To test for differences in learning rate among groups, we used a general linear model (glm) in R^107^, with poisson error structure, accounting for the fact that the session number at which the learning criterion was reached is a count variable, i.e. glm(sessions_to_LC ~ group, family=”poisson”), with 0, A, and B as the levels of the factor group, with 0 defining the reference group (intercept).

#### Statistical testing of individual response rates

To find out if an individual fish significantly responds in recall tests or unisensory performance tests, we counted how many of the $\:\nu\:\:$test trials elicited avoidance responses while the signal was being applied (correct positives, *CP*). For control, we counted how often the fish spontaneously crossed (false positives, *FP*) given $\:\nu\:{\prime\:}$ opportunities. For group 0, $\:\nu\:{\prime\:}$=10, i.e., 2 sham trials in each of the 5 test sessions. To obtain p-values, we used the proportion test implemented in the programming language R^107^, which compares the proportion of correct positives, ($\:CP/\nu\:$), with that of false positives, ($\:FP/\nu\:{\prime\:}$), using the following syntax: prop.test(c(*CP*, *FP)*, c($\:\nu\:,\nu\:{\prime\:})$, alternative=”two.sided”). For group A and B, rather than placing specific sham trials in the test sessions, we determined the spontaneous crossing rate during intertrial intervals. With ca. 20 intertrial intervals per session, of 120 s mean duration, the total number of opportunities (10 s intervals) in 10 test sessions is approximately given by 20 trials/session x 10 sessions x 120 s/10 sec = 1200, implying a small error margin for the estimated false positive rate *FPR*, defined as the number of spontaneous crossings divided by the number of crossing opportunities. We use the binomial test to compare the proportion of correct positives in test trials to the *FPR*, i.e., binomial.test(x = *CP*, n = $\:\nu\:$, p = *FPR*, alternative=”greater”).

#### Statistical testing of response rates at group level

To test for differences in response rates in LM: LM vs. LM: L tests, we used the following mixed-effects general linear model,

glmer.out <- lme4::glmer(cbind(*CP*,*N-CP*) ~ Condition + (1 | ID), data= …, family=”binomial”).

where glmer is from the R-package lme4^108^, *CP* is the number of correct positives, *N* is the number of trials (so that *CPR* = *CP/N*), the factor variable ‘Condition’ is either LM: LM or LM: L, and (1 | ID) is the random effect term, accounting for the fact that each individual zebrafish (factor variable ID) is tested under both conditions (paired test).

## Electronic supplementary material

Below is the link to the electronic supplementary material.

Supplementary Material 1.

Supplementary Material 2.

## Data Availability

Data is provided within the manuscript and supplementary information files.

## Appendix: Learning theory

In the following we show that the mathematical model of Rescorla and Wagner^50^ (loc. cit. RW) of classic (Pavlovian) conditioning fails to describe our experimental results. For the overshadowing scenario with two novel cues (A + X) in a reinforced compound CS (AX+), RW give the following differential equations for the incremental gain in association strength for cues A and X,

3 $$\frac{d}{{dt}}\lambda _{{\text{A}}} = \alpha _{{\text{A}}} \beta \left( {\lambda _{{{\text{AX}}}}^{\infty } - \lambda _{{{\text{AX}}}} \left( t \right)} \right),$$

4 $$\frac{d}{{dt}}\lambda _{{\text{X}}} = \alpha _{{\text{X}}} \beta \left( {\lambda _{{{\text{AX}}}}^{\infty } - \lambda _{{{\text{AX}}}} \left( t \right)} \right),$$

5 $$\lambda _{{{\text{AX}}}} \left( t \right) = \lambda _{{\text{A}}} \left( t \right) + \lambda _{{\text{X}}} \left( t \right),~$$

where $\:{\alpha\:}_{\text{A}}\ge\:0$ and $\:{\alpha\:}_{\text{X}}\ge\:0$ is the associability of cue A and X, resp., $\:\beta\:\le\:1$ is the US-specific learning rate, $\:{\lambda\:}_{\text{A}}\left(t\right)$, $\:{\lambda\:}_{\text{X}}\left(t\right),{\lambda\:}_{\text{A}\text{X}}\left(t\right)$ are the association strengths for the two components A, X, and the compound AX, resp., each after $\:t$ trials, and $\:{\lambda\:}_{\text{A}\text{X}}^{\infty\:,}$ is the asymptotic association strength for the compound. With the initial conditions $\:{\lambda\:}_{\text{A}}\left(0\right)={\lambda\:}_{\text{X}}\left(0\right)=0$ (i.e., naïve to A and X), Eqs. (3, 4) can be integrated to yield the following learning curves,

6 $$\lambda _{{\text{A}}} \left( t \right) = \left( {\frac{{\alpha _{{\text{A}}} }}{{\alpha _{{\text{A}}} + \alpha _{{\text{X}}} }}} \right)\lambda _{{{\text{AX}}}}^{\infty } ~\left( {1 - \exp \left( { - \left( {\alpha _{{\text{A}}} + \alpha _{{\text{X}}} } \right)\beta t} \right)~} \right),$$

7 $$\lambda _{{\text{X}}} \left( t \right) = \left( {\frac{{\alpha _{{\text{X}}} }}{{\alpha _{{\text{A}}} + \alpha _{{\text{X}}} }}} \right)\lambda _{{{\text{AX}}}}^{\infty } ~\left( {1 - \exp \left( { - \left( {\alpha _{{\text{A}}} + \alpha _{{\text{X}}} } \right)\beta t} \right)~} \right).$$

Thus, the two novel cues compete with one another for gain in association strength according to their relative salience, $\:\frac{{\alpha\:}_{A}}{{\alpha\:}_{X}+{\alpha\:}_{A}}$ versus $\:\frac{{\alpha\:}_{X}}{{\alpha\:}_{X}+{\alpha\:}_{A}}$. Note that Eqs. (6) and (7) predict a simple exponential learning curve. In terms of the more general Weibull function (Eq. 2), the solutions of the RW-equations (Eqs. 6, 7) have shape parameter $\:S=1$ and scale parameter $\:1/L=\beta\:\left({\alpha\:}_{\text{X}}+{\alpha\:}_{\text{A}}\right)$. The various learning curves we observed in our experiments can be fitted best with Weibull functions of either hyperbolic $\:(0<S<1)$ or sigmoidal $\:(S>1)$ shape. While this mismatch is not serious (and could be fixed with a time dependence of the learning rate), the major discrepancy concerns the asymptotic association strengths predicted by the RW model (Eqs. 6, 7), which for L, M, and LM can be written as

8 $$\lambda _{L}^{\infty } = \frac{{\alpha _{{\text{L}}} }}{{\alpha _{{\text{M}}} + \alpha _{{\text{L}}} }}\lambda _{{{\text{LM}}}}^{\infty } ~,~~\lambda _{M}^{\infty } = \frac{{\alpha _{{\text{M}}} }}{{\alpha _{{\text{M}}} + \alpha _{{\text{L}}} }}~\lambda _{{{\text{LM}}}}^{\infty } ~,~~~~\lambda _{{{\text{LM}}}}^{\infty } ~ = \lambda _{{\text{L}}}^{\infty } ~ + \lambda _{{\text{M}}}^{\infty } ~,$$

Where $\:{\alpha\:}_{L}\ge\:0$ and $\:{\alpha\:}_{\text{M}}\ge\:0$ is the specific learning rate for the visual and magnetic cue, respectively. In essence, Eq. (8) predicts $\:{\lambda\:}_{\text{L}\text{M}}^{\infty\:}\ge\:{\lambda\:}_{L}^{\infty\:}\:$, i.e., the response rate in LM: LM tests should be at least as high as in LM: L tests, while we observed the opposite in group B fish, which would imply $\:{\lambda\:}_{\text{M}}^{\infty\:}<0.\:$We cannot directly determine $\:{\lambda\:}_{\text{M}}^{\infty\:}$ from the correct positive response rate in LM: M tests, because it is statistically indistinguishable from the false positive rate, *FPR* (floor effect). However, we may attempt to estimate $\:{\alpha\:}_{\text{M}}/{\alpha\:}_{\text{L}}$ and $\:{\lambda\:}_{\text{M}}^{\infty\:}\:$indirectly via Eq. (8), by taking the correct response rates in LM: LM trials and LM: L trials (Table 2) as $\:{\lambda\:}_{\text{L}\text{M}}^{\infty\:}$ and $\:{\lambda\:}_{\text{L}}^{\infty\:}$, respectively. That approach yields

9 $${\text{for}}\;{\text{group}}\;{\text{A:}} \quad \frac{{\alpha _{{\text{M}}} }}{{\alpha _{{\text{L}}} }} \approx 0, \quad \lambda _{{\text{M}}}^{\infty } = 0,\quad \lambda _{{\text{L}}}^{\infty } = \lambda _{{{\text{LM}}}}^{\infty } \; ,$$

and

10 $${\text{for}}\;{\text{group}}\;{\text{B:}}\quad \frac{{\alpha _{{\text{M}}} }}{{\alpha _{{\text{L}}} }} \approx - \frac{1}{{12}},\quad \lambda _{{\text{M}}}^{\infty } = - \frac{1}{{11}}\lambda _{{{\text{LM}}}}^{\infty } ,\quad \lambda _{{\text{L}}}^{\infty } = \frac{{12}}{{11}}\lambda _{{{\text{LM}}}}^{\infty } \; .$$

The last result is at odds with the RW assumption of $\:{\alpha\:}_{\text{M}}\:$and $\:{\alpha\:}_{\text{L}}\:$being both non-negative. From a pure mathematical standpoint, we can relax the non-negativity constraint somewhat (on condition that the sum $\:{\alpha\:}_{\text{M}}+{\alpha\:}_{\text{L}}\:$be positive, which is certainly fulfilled for $\:{\alpha\:}_{\text{M}}/{\alpha\:}_{\text{L}}\approx\:-1/12\;$). But even when allowing $\:{\alpha\:}_{\text{M}}$ to be slightly negative, we are still left with a negative value of $\:{\lambda\:}_{\text{M}}^{\infty\:}$ (“inhibition”) and that in turn would imply a significantly lower response rate in LM: M compared to the *FPR*, which was not observed either. Put differently, a negative $\:{\alpha\:}_{\text{M}}$ would adequately describe the distractive effect of M in the compound LM, but at the same time leads to an incorrect prediction for the M component alone. Thus, if a self-consistent prediction from a model is wrong, then the model is simply not applicable to the situation at hand. This is due most likely to the RW assumption that the US-specific learning rate $\:\beta\:$ and cue-specific learning parameters $\:{\alpha\:}_{\text{X}}\:$, $\:{\alpha\:}_{\text{A}\:}\:$are all independent of one another (Eq. 3, 4). However, this assumption appears to be critically violated in cases where one cue is clearly favored over another in a given learning context, as here and in the illness-induced aversions mentioned in the Discussion: Salience of the visual signal (see also^51^, therein Failure 9 of RW: cue-to-consequence effects and Cluster 3: Fixed associability (i.e., $\:\alpha\:$ and $\:\beta\:$) of a stimulus).
