# Supplementary material for: Extremely low frequency magnetic field distracts zebrafish from a visual cognitive task
Source: Sci Rep. 2025 Mar 12;15:8589. doi: 10.1038/s41598-025-90194-x (PMC11903689; doi:10.1038/s41598-025-90194-x)
Supplement: Supplementary file 1 — Supplementary Material 1. [file 41598_2025_90194_MOESM1_ESM.zip › ZIP/41598_2025_90194_MOESM7_ESM.html]

Ziegenbalg et al: EMF distracts zebrafish. Tables 2/3/4 - Statistical Treatment


Code 

- Show All Code
- Hide All Code
- Download Rmd

# Ziegenbalg et al: EMF distracts zebrafish. Tables 2/3/4 - Statistical Treatment

# Table 2, group A: p values CPR(LM:M)>FPR


```
m13=binom.test(4,13,p=0.27,alternative='greater')
m14=binom.test(2,12,p=0.19,alternative='greater')
m11=binom.test(3,12,p=0.33,alternative='greater')

cbind(lapply(list(m13,m14,m11),function(x) return(x$p.value) ))
```


```
     [,1]     
[1,] 0.4825762
[2,] 0.6957058
[3,] 0.8124391
```

# Table 2, group B: p values CPR(LM:M)>FPR


```
m17=binom.test(6,15,p=0.35,alternative='greater')
m15=binom.test(0,17,p=0.14,alternative='greater')
m18=binom.test(5,14,p=0.36,alternative='greater')
m19=binom.test(4,15,p=0.2,alternative='greater')
m16=binom.test(4,16,p=0.06,alternative='greater')

cbind(lapply(list(m17,m15,m18,m19,m16),function(x) return(x$p.value) ))
```


```
     [,1]      
[1,] 0.4357179 
[2,] 1         
[3,] 0.6079885 
[4,] 0.3518379 
[5,] 0.01316628
```


#for comparison, CP(LM:L) response rates are \*\*\* significantly higher
than FPR


```
#e.g. ZF15
binom.test(14,18,p=0.14,alternative='greater')
```


```
    Exact binomial test

data:  14 and 18
number of successes = 14, number of trials = 18,
p-value = 1.943e-09
alternative hypothesis: true probability of success is greater than 0.14
95 percent confidence interval:
 0.5611172 1.0000000
sample estimates:
probability of success 
             0.7777778
```

# Table 3: Sessions to Learning Criterion (Between groups)


```
sessions_to_LC=c(8,7,7,
     7,15,7,9,15,
     9,6,7,8.5,4)
grp=c("LM_A","LM_A","LM_A",
      "LM_B","LM_B","LM_B","LM_B","LM_B",
      "L_0","L_0","L_0","L_0","L_0")
  
dfr=data.frame(LC=sessions_to_LC,group=grp)
glmP.out=glm(LC~group,data=dfr,family='poisson')
```


```
Warning: non-integer x = 8.500000
```


```
summary(glmP.out)
```


```
Call:
glm(formula = LC ~ group, family = "poisson", data = dfr)

Deviance Residuals: 
    Min       1Q   Median       3Q      Max  
-1.1992  -0.5047  -0.1240   0.5876   1.2712  

Coefficients:
            Estimate Std. Error z value Pr(>|z|)    
(Intercept)  1.93152    0.17025  11.345   <2e-16 ***
groupLM_A    0.06091    0.27284   0.223   0.8233    
groupLM_B    0.42933    0.21875   1.963   0.0497 *  
---
Signif. codes:  
0 ‘***’ 0.001 ‘**’ 0.01 ‘*’ 0.05 ‘.’ 0.1 ‘ ’ 1

(Dispersion parameter for poisson family taken to be 1)

    Null deviance: 13.357  on 12  degrees of freedom
Residual deviance:  8.848  on 10  degrees of freedom
AIC: Inf

Number of Fisher Scoring iterations: 4
```


```
#Percentage change in LC for LM_B vs L_0
(exp(glmP.out$coefficients['groupLM_B'])-1)*100
```


```
groupLM_B 
 53.62319
```

# Table 4

# LM:LM recall vs LM:L response rates for group B

# Within group B, between test conditions


```
IDs=c('z15','z16','z17','z18','z19')
CSgo=c(151,156,147,114,114) 
CSnogo=c(41,34,15,31,28) 
Lgo=c(14,20,28,21,24)
Lnogo=c(4,1,0,2,2)


dfr=data.frame(cond='LM:LM',CP=CSgo,FN=CSnogo,id=IDs )
dfr=rbind(dfr,
  data.frame(cond='LM:L',CP=Lgo,FN=Lnogo,id=c('z15','z16','z17','z18','z19') )
)

dfr$rate=dfr$CP/(dfr$FN+dfr$CP)
dfr$odds=dfr$rate/(1-dfr$rate)
dfr$logodd=log(dfr$odds)

#aggregate(cbind(rate,odds,logodd) ~ cond,data=dfr,FUN=mean)
```


```
library(lme4)
```


```
Loading required package: Matrix
```


```
mixB.out<-lme4::glmer(cbind(CP, FN) ~ cond + (1 | id) ,family = binomial, data = dfr)

summary(mixB.out)->s 

print(s)
```


```
Generalized linear mixed model fit by maximum likelihood
  (Laplace Approximation) [glmerMod]
 Family: binomial  ( logit )
Formula: cbind(CP, FN) ~ cond + (1 | id)
   Data: dfr

     AIC      BIC   logLik deviance df.resid 
    57.3     58.3    -25.7     51.3        7 

Scaled residuals: 
     Min       1Q   Median       3Q      Max 
-1.82586 -0.22402 -0.00092  0.41184  1.19998 

Random effects:
 Groups Name        Variance Std.Dev.
 id     (Intercept) 0.09391  0.3064  
Number of obs: 10, groups:  id, 5

Fixed effects:
            Estimate Std. Error z value Pr(>|z|)    
(Intercept)   2.4871     0.3738   6.655 2.84e-11 ***
condLM:LM    -0.9340     0.3594  -2.599  0.00935 ** 
---
Signif. codes:  
0 ‘***’ 0.001 ‘**’ 0.01 ‘*’ 0.05 ‘.’ 0.1 ‘ ’ 1

Correlation of Fixed Effects:
          (Intr)
condLM:LM -0.898
```


```
logit <- function(ratio) { 
 log(ratio/(1-ratio))}

expit <- function(li) { 
  1/(1+exp(-li))
} # yields ratio
```

# Mean response rates


```
# MEAN LM:L 
rL=expit(s$coefficients[1,1]) 
# MEAN LM:LM 
rLM=expit(s$coefficients[1,1]+s$coefficients[2,1]) 

OR=(rL/(1-rL))/(rLM/(1-rLM))

# directly: OR=1/exp(s$coefficients[2,1])

print(paste("CPR (LM:L) :",rL))
```


```
[1] "CPR (LM:L) : 0.923234905230892"
```


```
print(paste("CPR (LM:LM):",rLM))
```


```
[1] "CPR (LM:LM): 0.825366479312474"
```


```
print(paste("Odds Ratio:",OR))
```


```
[1] "Odds Ratio: 2.54465674818237"
```

LS0tCnRpdGxlOiAiWmllZ2VuYmFsZyBldCBhbDogRU1GIGRpc3RyYWN0cyB6ZWJyYWZpc2guIFRhYmxlcyAyLzMvNCAtIFN0YXRpc3RpY2FsIFRyZWF0bWVudCIKb3V0cHV0OiBodG1sX25vdGVib29rCi0tLQoKIyBUYWJsZSAyLCBncm91cCBBOiBwIHZhbHVlcyBDUFIoTE06TSk+RlBSIApgYGB7cn0KCm0xMz1iaW5vbS50ZXN0KDQsMTMscD0wLjI3LGFsdGVybmF0aXZlPSdncmVhdGVyJykKbTE0PWJpbm9tLnRlc3QoMiwxMixwPTAuMTksYWx0ZXJuYXRpdmU9J2dyZWF0ZXInKQptMTE9Ymlub20udGVzdCgzLDEyLHA9MC4zMyxhbHRlcm5hdGl2ZT0nZ3JlYXRlcicpCgpjYmluZChsYXBwbHkobGlzdChtMTMsbTE0LG0xMSksZnVuY3Rpb24oeCkgcmV0dXJuKHgkcC52YWx1ZSkgKSkKCgpgYGAKCiMgVGFibGUgMiwgZ3JvdXAgQjogcCB2YWx1ZXMgQ1BSKExNOk0pPkZQUiAKYGBge3J9CgptMTc9Ymlub20udGVzdCg2LDE1LHA9MC4zNSxhbHRlcm5hdGl2ZT0nZ3JlYXRlcicpCm0xNT1iaW5vbS50ZXN0KDAsMTcscD0wLjE0LGFsdGVybmF0aXZlPSdncmVhdGVyJykKbTE4PWJpbm9tLnRlc3QoNSwxNCxwPTAuMzYsYWx0ZXJuYXRpdmU9J2dyZWF0ZXInKQptMTk9Ymlub20udGVzdCg0LDE1LHA9MC4yLGFsdGVybmF0aXZlPSdncmVhdGVyJykKbTE2PWJpbm9tLnRlc3QoNCwxNixwPTAuMDYsYWx0ZXJuYXRpdmU9J2dyZWF0ZXInKQoKY2JpbmQobGFwcGx5KGxpc3QobTE3LG0xNSxtMTgsbTE5LG0xNiksZnVuY3Rpb24oeCkgcmV0dXJuKHgkcC52YWx1ZSkgKSkKCgoKYGBgCgojZm9yIGNvbXBhcmlzb24sIENQKExNOkwpIHJlc3BvbnNlIHJhdGVzIGFyZSAqKiogc2lnbmlmaWNhbnRseSBoaWdoZXIgdGhhbiBGUFIgIApgYGB7cn0KI2UuZy4gWkYxNQpiaW5vbS50ZXN0KDE0LDE4LHA9MC4xNCxhbHRlcm5hdGl2ZT0nZ3JlYXRlcicpCgpgYGAKCiMgVGFibGUgMzogU2Vzc2lvbnMgdG8gTGVhcm5pbmcgQ3JpdGVyaW9uIChCZXR3ZWVuIGdyb3VwcykKYGBge3J9CgpzZXNzaW9uc190b19MQz1jKDgsNyw3LAogICAgIDcsMTUsNyw5LDE1LAogICAgIDksNiw3LDguNSw0KQpncnA9YygiTE1fQSIsIkxNX0EiLCJMTV9BIiwKICAgICAgIkxNX0IiLCJMTV9CIiwiTE1fQiIsIkxNX0IiLCJMTV9CIiwKICAgICAgIkxfMCIsIkxfMCIsIkxfMCIsIkxfMCIsIkxfMCIpCiAgCmRmcj1kYXRhLmZyYW1lKExDPXNlc3Npb25zX3RvX0xDLGdyb3VwPWdycCkKZ2xtUC5vdXQ9Z2xtKExDfmdyb3VwLGRhdGE9ZGZyLGZhbWlseT0ncG9pc3NvbicpCnN1bW1hcnkoZ2xtUC5vdXQpCgpgYGAKCmBgYHtyfQoKI1BlcmNlbnRhZ2UgY2hhbmdlIGluIExDIGZvciBMTV9CIHZzIExfMAooZXhwKGdsbVAub3V0JGNvZWZmaWNpZW50c1snZ3JvdXBMTV9CJ10pLTEpKjEwMAoKYGBgCgojIFRhYmxlIDQgCiMgTE06TE0gcmVjYWxsIHZzIExNOkwgcmVzcG9uc2UgcmF0ZXMgZm9yIGdyb3VwIEIgCiMgV2l0aGluIGdyb3VwIEIsIGJldHdlZW4gdGVzdCBjb25kaXRpb25zCmBgYHtyfSAKCklEcz1jKCd6MTUnLCd6MTYnLCd6MTcnLCd6MTgnLCd6MTknKQpDU2dvPWMoMTUxLDE1NiwxNDcsMTE0LDExNCkgCkNTbm9nbz1jKDQxLDM0LDE1LDMxLDI4KSAKTGdvPWMoMTQsMjAsMjgsMjEsMjQpCkxub2dvPWMoNCwxLDAsMiwyKQoKCmRmcj1kYXRhLmZyYW1lKGNvbmQ9J0xNOkxNJyxDUD1DU2dvLEZOPUNTbm9nbyxpZD1JRHMgKQpkZnI9cmJpbmQoZGZyLAogIGRhdGEuZnJhbWUoY29uZD0nTE06TCcsQ1A9TGdvLEZOPUxub2dvLGlkPWMoJ3oxNScsJ3oxNicsJ3oxNycsJ3oxOCcsJ3oxOScpICkKKQoKZGZyJHJhdGU9ZGZyJENQLyhkZnIkRk4rZGZyJENQKQpkZnIkb2Rkcz1kZnIkcmF0ZS8oMS1kZnIkcmF0ZSkKZGZyJGxvZ29kZD1sb2coZGZyJG9kZHMpCgojYWdncmVnYXRlKGNiaW5kKHJhdGUsb2Rkcyxsb2dvZGQpIH4gY29uZCxkYXRhPWRmcixGVU49bWVhbikKCmBgYAoKYGBge3J9CgpsaWJyYXJ5KGxtZTQpCgptaXhCLm91dDwtbG1lNDo6Z2xtZXIoY2JpbmQoQ1AsIEZOKSB+IGNvbmQgKyAoMSB8IGlkKSAsZmFtaWx5ID0gYmlub21pYWwsIGRhdGEgPSBkZnIpCgpzdW1tYXJ5KG1peEIub3V0KS0+cyAKCnByaW50KHMpCgpsb2dpdCA8LSBmdW5jdGlvbihyYXRpbykgeyAKIGxvZyhyYXRpby8oMS1yYXRpbykpfQoKZXhwaXQgPC0gZnVuY3Rpb24obGkpIHsgCiAgMS8oMStleHAoLWxpKSkKfSAjIHlpZWxkcyByYXRpbwoKCgpgYGAKIyBNZWFuIHJlc3BvbnNlIHJhdGVzIApgYGB7cn0KCiMgTUVBTiBMTTpMIApyTD1leHBpdChzJGNvZWZmaWNpZW50c1sxLDFdKSAKIyBNRUFOIExNOkxNIApyTE09ZXhwaXQocyRjb2VmZmljaWVudHNbMSwxXStzJGNvZWZmaWNpZW50c1syLDFdKSAKCk9SPShyTC8oMS1yTCkpLyhyTE0vKDEtckxNKSkKCiMgZGlyZWN0bHk6IE9SPTEvZXhwKHMkY29lZmZpY2llbnRzWzIsMV0pCgpwcmludChwYXN0ZSgiQ1BSIChMTTpMKSA6IixyTCkpCnByaW50KHBhc3RlKCJDUFIgKExNOkxNKToiLHJMTSkpCnByaW50KHBhc3RlKCJPZGRzIFJhdGlvOiIsT1IpKQoKYGBgCg==
