# Supplementary material for: Extremely low frequency magnetic field distracts zebrafish from a visual cognitive task
Source: Sci Rep. 2025 Mar 12;15:8589. doi: 10.1038/s41598-025-90194-x (PMC11903689; doi:10.1038/s41598-025-90194-x)

SUPPLEMENTARY MATERIAL

Extremely low frequency magnetic field distracts zebrafish from a visual cognitive task

Laura Ziegenbalg[1] (ORCID ID 0000-0002-2320-4431)
Onur Güntürkin[2] (ORCID ID 0000-0003-4173-5233)
Michael Winklhofer[1,3,*] (ORCID ID 0000-0003-1352-9723)

[1] AG Sensory Biology of Animals, Institute for Biology and Environmental Sciences, Carl von Ossietzky Universität Oldenburg, Oldenburg, 26129, Germany.

[2] Department of Biopsychology, Faculty of Psychology, Ruhr University Bochum, Universitätsstraße 150, D-44780 Bochum, Germany.

[3] Research Center Neurosensory Science, Carl von Ossietzky University of Oldenburg, Oldenburg, 26111, Germany.

[*]To whom correspondence should be addressed: michael.winklhofer@uol.de

Supplementary Statistics:

StatisticsTables2-4.Rmd (also in html format): R-Notebook containing all statistical analysis reported in the paper

Supplementary Data Files

L.xslx: Data for fish of group 0 (L+ training data, L:L recall tests)

LM_A_15uT.xlsx: Data for fish of group A (LM+ training data, with M=0.015 mT)

LM_B_60uT.xlsx: Data for fish of group B (LM+ training data, with M=0.06 mT)

Recall_SplitCue_Tests_LM_A_15uT.xlsx (Data for fish of group A, tested under LM:LM, LM:L, LM:M)

Recall_SplitCue_Tests_LM_B_15uT.xlsx (Data for fish of group B, tested under LM:LM, LM:L, LM:M)

Supplementary Figures (next pages)

SI Figure 1 Setup

SI Figure 2 Example trajectory, to show the principle of false-positive rate detection

SI Figure 3 Magnetic field exposure

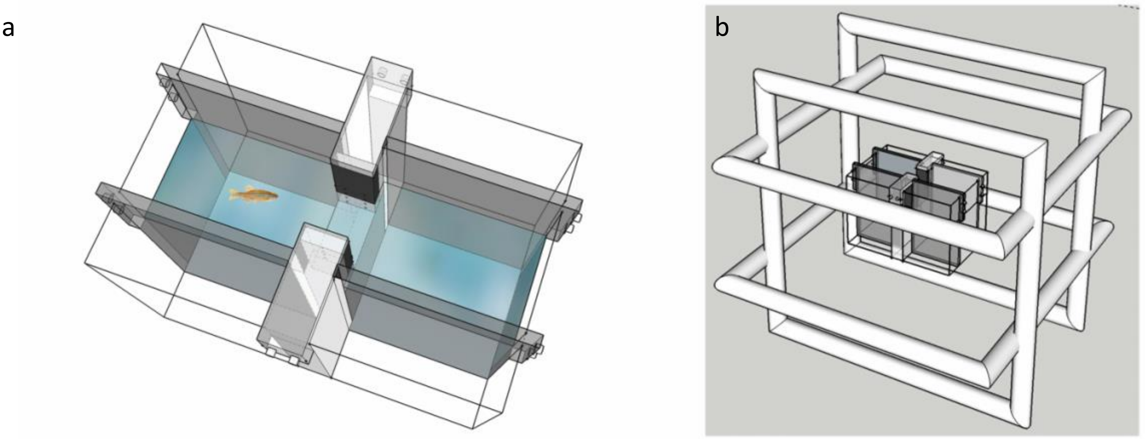

SI Figure 1 a) Home-built fully automated Horner-type shuttlebox, consisting of two compartments (each 13 cm × 12 cm × 20 cm height), separated by a hurdle separated by a hurdle (5 cm wide, 3 cm long, and 11 cm high) submersed 1 cm deep below the water surface. A light barrier detects the shuttling of a fish between the compartments. b) Placement of the setup in a two-axis square Helmholtz-coil system with double wrapped coils. The two horizontal coil frames coils are energized with a sinusoidally oscillating current throughout each session, but only deliver a magnetic field stimulus when the current flows parallel in the two windings. Otherwise, the two windings carry the same current as in exposure, but in antiparallel direction so that two magnetic fields compensate each other to produce no net magnetic field.

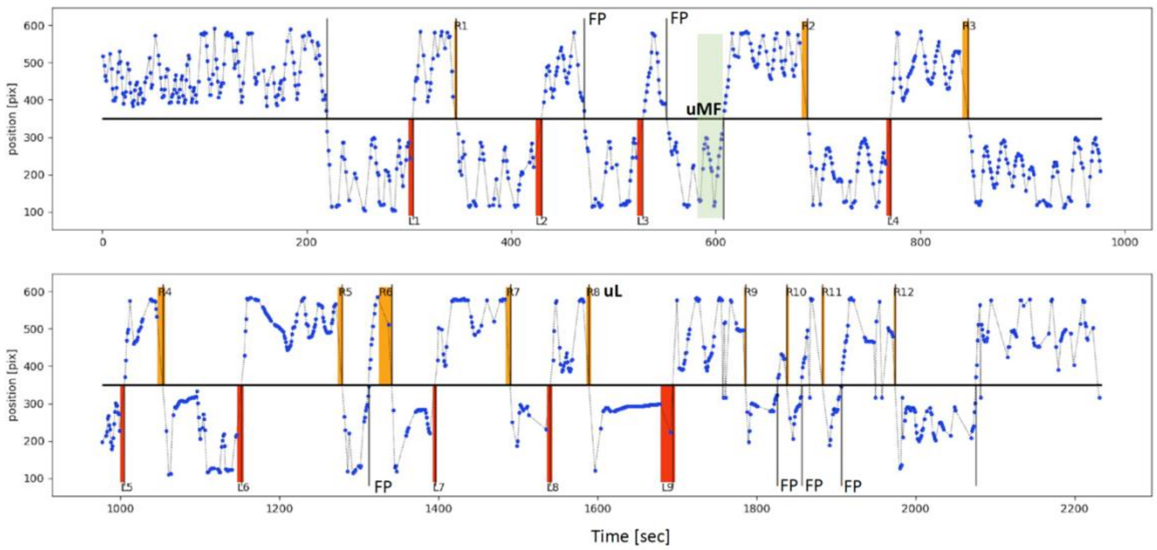

SI Figure 2. One-dimensional fish trajectory extracted from a video sequence recording a test session (after LM+). The dots show the position of the fish along the long axis of the shuttle box as a function of time. The solid lines connecting the dots is a guide to the eye. The position of the hurdle is indicated by the horizontal black line (at 350 pixels). The LED-on events are marked in color (red or orange, depending on the side where the fish was when the light went on, hence the labels L and R for left and right) and the width of the color bar indicates the trial length. In this example shown here (ZF15, group B, test session 4), the session comprised 20 CS recall trials (LM:LM, indicated by L1-L9, R1-R7, R9-R12), one unisensory LM:L trial (**uL**, R8 at t=1586 sec, crossing within 10 sec, i.e. correct positive), and one unisensory LM:M trial (**uMF**, green box at t=575 sec, which did not elicit crossing within 10 sec, i.e., false negative). The multimodal CS elicited 17 correct positive responses (within 10 sec) in 20 trials, with three false negatives at R4, R6, and L9, where the fish did not cross within 10 sec after onset of cue presentation. Conversely, spontaneous crossings when no stimulus was presented count as false positives (indicated here as black vertical bars labeled FP). In this example, we identified 6 FP between the first trial (L1) and the last trial (R12). To obtain the FP rate, we need to be put the number of FP in relation to the total number of crossing opportunities the fish had in intertrial intervals. Dividing the total session time (1670 sec between L1 and R12) in 10 second long subintervals, we obtain 167 (cue + non-cue) trials, of which 22 were true trials with a cue presented, thus yielding 145 crossing opportunities (non-cue trials) and a false positive rate of 6/145=0.04. The fish was identifiable in 65% of all frames (frame rate was 25 frames per second). For recorded magnetometer trace, see SI Figure 2 on next page.

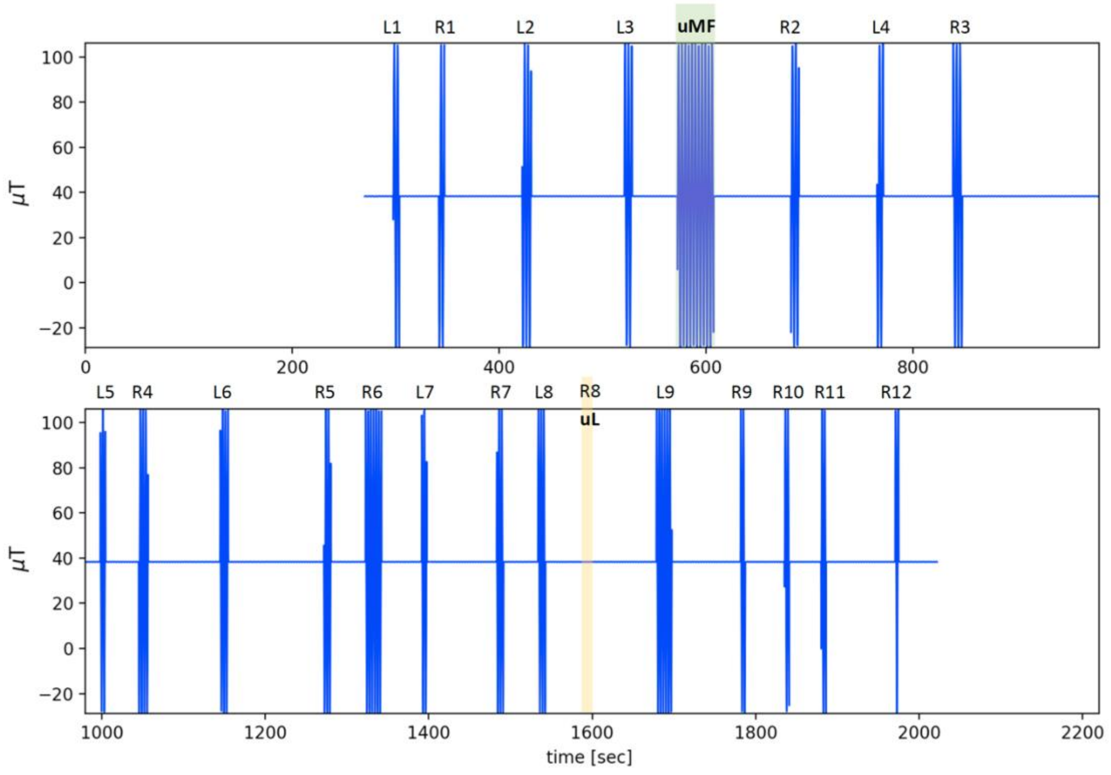

SI Figure 3. Magnetometer recording of the vertical magnetic field component during the test session shown in SI Figure 2 (recording started 25 sec before first trial and ended ca. 50 sec after the onset of the last trial). The unisensory LM:M trial (**uMF**) is highlighted in green, the unisensory LM:L trial (**uL**) is highlighted in yellow. The magnetic field oscillates about the ambient magnetic field at a rate of 0.3 Hz (i.e. 3 full cycles in 10 sec). The oscillation amplitude is 60 μT (0.06 mT, “MF exposure mode”) during LM:LM trials (R_i and L_j) as well as LM:M trial, and 0.1 microTesla (100 nT, “sham MF mode”) during intertrials and LM:L trials (see zoom below).

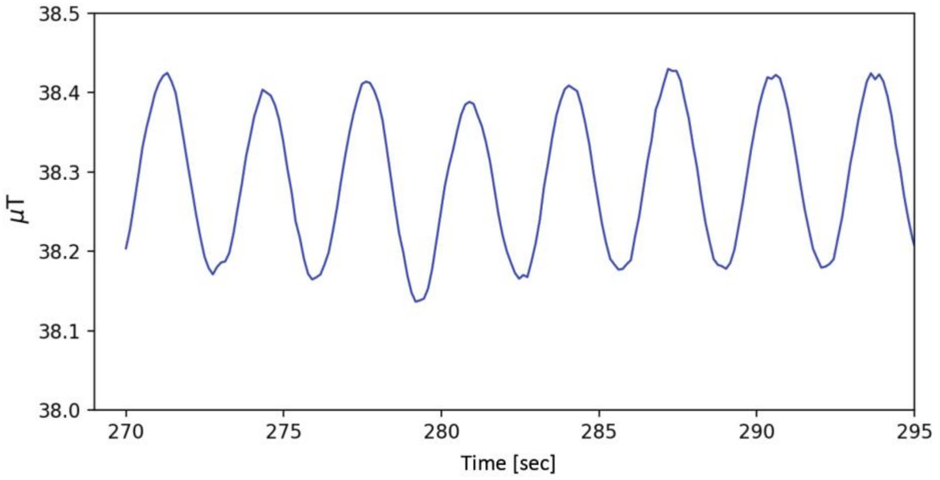

Supplement: Supplementary file 2 — Supplementary Material 2. [file 41598_2025_90194_MOESM2_ESM.pdf]
